# Economic Evaluation of Digital Health Interventions in Palliative Care: A Systematic Review of the Literature

**DOI:** 10.3389/fdgth.2021.730755

**Published:** 2021-11-03

**Authors:** Panagiota Naoum, Elpida Pavi, Kostas Athanasakis

**Affiliations:** Laboratory for Health Technology Assessment (LabHTA), Department of Public Health Policy, University of West Attica, Athens, Greece

**Keywords:** digital health, economic evaluation, costs and outcomes, palliative care, systematic review

## Abstract

**Introduction:** Digital health interventions can facilitate the provision of palliative care. However, the economic evaluation of such interventions has not yet been a standard practice. The present study aimed to identify the existing literature on the particular subject.

**Methods:** A systematic search was conducted in six literature databases between 2010 and 2021: PubMed, Scopus, DARE, NHS EED, Cochrane Database of Systematic Reviews, and Cochrane Central Register of Controlled Trials. Methodological quality was assessed with the Drummond Checklist.

**Results:** The search identified 423 publications, 66 of which were removed as duplicates, resulting in 357 records to be screened by title and abstract. Ten studies were subjected to full-text review and 3 were included in the analysis. The interventions of these studies referred to video consultations and eHealth interventions for symptom management. Overall, the digital health interventions incurred lower costs compared with usual care or no intervention and were considered cost saving and cost-effective. The methodological quality of the studies was considered good.

**Conclusion:** The results of this systematic review indicate that the use of digital health interventions has the potential to be cost-effective in palliative care. However, applicability and generalizability of the evidence is uncertain, mainly due to methodological heterogeneity and scarcity of research.

## Introduction

Palliative care is defined as specialized medical care for people with a serious illness. This type of care is focused on providing relief from the symptoms and stress of the illness (1). In this context, palliative care aims to improve quality of life (QoL) both for the patient and the family. Early access to palliative care is currently a recommended standard practice for patients, particularly those with advanced-stage and/or incurable disease and/or a substantial symptom burden and/or compromised physical status, while it can also be used for symptom management in not life-threatening and curable diseases (2). Additionally, palliative care extends beyond the relief of physical symptoms as it seeks to strengthen the psychological, spiritual, and social domains in order to provide greater comfort to patients (3).

Advances in medical technology have facilitated improvements in the assessment of a symptom's progress in order to appropriately address its management. Digital health (or ehealth) has been defined as “an emerging field in the interaction of medical informatics, public health and business, referring to health services and information delivered or enhanced through the Internet and related technologies. In a broad sense, the term characterizes not only a technical development, but also a state-of-mind, a way of thinking, an attitude, and a commitment for networked, global thinking, to improve health care locally, regionally, and worldwide by using information and communication technology” (4). Additionally to this, the World Health Organization has defined digital health interventions as “a discrete functionality of digital technology that is applied to achieve health objectives and is implemented within digital health applications and ICT systems, including communication channels such as text messages” (5).

In palliative care, digital health interventions are used to bridge the gap between healthcare professionals and patients with serious illness, by narrowing the time between the manifestation of a symptom and its reporting, thus, facilitating its prompt management. Moreover, some more recent eHealth applications may even allow for self-management of symptoms when appropriate. The effectiveness and applicability of such interventions have been a research interest in the last decades, the results of which indicate positive outcomes (6, 7).

Although digital health interventions are generally assumed to be cost-effective or cost-saving (8), there is not sufficient evidence in the scientific literature to support this. In particular, there have been only a few systematic reviews on the cost-effectiveness of telemedicine and digital health (9, 10), however, none of these report findings on palliative care. The economic evaluation of digital health interventions is important as it aims to inform decision makers on their relative value for money compared to specific alternatives.

In this context, the objective of this paper was to perform a systematic review with the aim to identify and critically assess published studies on the economic evaluation of digital health interventions in the setting of palliative care.

## Methods

The methodology of the present systematic review followed the Preferred Reporting Items for Systematic Reviews and Meta-Analyses (PRISMA) statement (11).

### Search Strategy

The search strategy of the analysis was applied in the six following databases: PubMed, SCOPUS, Database of Abstracts of Reviews of Effects (DARE), NHS Economic Evaluation Database (NHS EED), Cochrane Database of Systematic Reviews, and Cochrane Central Register of Controlled Trials. The timeframe defined for the systematic review of the literature was from January 2010 to May 8th, 2021. The timeframe of the search was selected based on an initial search in the literature for relevant publications in the field, in view of the rapid developments in digital health technology. Additional records were retrieved through reference scanning.

The concept of palliative care is based on the relief of symptoms and stress from the underlying disease and/or its treatment (12). Therefore, the search process took into consideration any intervention aiming to improve quality of life including symptom management.

The search strategy was formed to include the terms “economic evaluation” + “digital health” + “palliative care” as well as their synonyms and relevant terms and MeSH terms, adjusted accordingly for each database. In order to optimize sensitivity and specificity of the used key words, the respective search guidelines of each database and published systematic reviews on relevant topics were consulted. An example of the full Scopus database search and the terms used is presented in Table 1.

**Table 1:** Example of full search.

#1	TITLE-ABS-KEY({economic evaluation})	32,070
#2	TITLE-ABS-KEY({cost-benefit})	110,366
#3	TITLE-ABS-KEY({cost-utility})	5,019
#4	TITLE-ABS-KEY({cost-effectiveness})	75,084
#5	TITLE-ABS-KEY({cost-effective})	206,357
#6	TITLE-ABS-KEY({cost consequences})	372
#7	TITLE-ABS-KEY(“cost minimi?ation”)	9,650
#8	TITLE-ABS-KEY({costs and benefits})	20,442
#9	TITLE-ABS-KEY({health care costs})	49,130
#10	#1 OR #2 OR #3 OR #4 OR #5 OR #6 OR #7 OR #8 OR #9	415,592
#11	TITLE-ABS-KEY(“Palliat*”)	145,398
#12	TITLE-ABS-KEY(“Hospice*”)	23,788
#13	TITLE-ABS-KEY(“terminal care”)	38,534
#14	TITLE-ABS-KEY(“end of life”)	42,301
#15	TITLE-ABS-KEY(“EOL care”)	1,146
#16	#11 OR #12 OR #13 OR #14 OR #15	197,144
#17	INDEXTERMS(telemedicine)	38,918
#18	TITLE-ABS-KEY(telehealth)	13,654
#19	TITLE-ABS-KEY(mhealth)	13,156
#20	TITLE-ABS-KEY({m-health})	1,982
#21	TITLE-ABS-KEY(ehealth)	10,231
#22	TITLE-ABS-KEY({e-health})	10,137
#23	TITLE-ABS-KEY({electronic health})	41,854
#24	TITLE-ABS-KEY({digital health})	3,765
#25	TITLE-ABS-KEY(video)	502,881
#26	TITLE-ABS-KEY(phone)	134,831
#27	TITLE-ABS-KEY(ipad)	4,360
#28	TITLE-ABS-KEY(tablet)	127,309
#29	TITLE-ABS-KEY(“text messag*”)	12,126
#30	TITLE-ABS-KEY(email)	29,571
#31	TITLE-ABS-KEY(sms)	129,858
#32	TITLE-ABS-KEY(web)	584,923
#33	#17 OR #18 OR #19 OR #20 OR #21 OR #22 OR #23 OR #24 OR #25 OR #26 OR #27 OR #28 OR #29 OR #30 OR #31	1,548,647
#34	#10 AND #16 AND #33	121
#35	#34 AND PUBYEAR > 2009	100


The eligibility of the identified records for inclusion in the analysis was judged based on the following criteria:

- Population: any population group that could require palliative care (including palliative care models, symptom management interventions, etc).
- Intervention: any kind of digital health intervention that facilitates the provision/outcomes of palliative care.
- Comparison: Palliative care vs. usual care or comparison between different palliative care interventions.
- Outcomes: economic evaluation (including: cost-effectiveness, cost-utility, cost minimization, etc).

Criteria for exclusion from the analysis were: studies which do not estimate costs relative to outcomes, studies with no comparator, studies focusing on caregivers, studies comparing palliative care with cure, publications on case reports/case studies, animal studies. Language was not considered an exclusion criterion.

### Data Extraction & Analysis

Screening was conducted by two of the authors (PN and KA), separately. Any disputes in the screening process between the two reviewers was resolved by the third author, acting as an independent reviewer. The identified records were screened by title and abstract and, after the initial screening, full texts were retrieved and reviewed. Study protocols, pilot, and feasibility studies were not included for the review, but were used as other sources to search for potential relevant publications with their results.

Upon selection of the included studies, data were extracted by one author (PN) and corroborated by a second author (EP). The results section employs a narrative synthesis approach. All studies included in the final analysis were evaluated for their methodological quality using the Drummond Checklist for assessing economic evaluations (13).

## Results

### Overview

**Figure 1:**
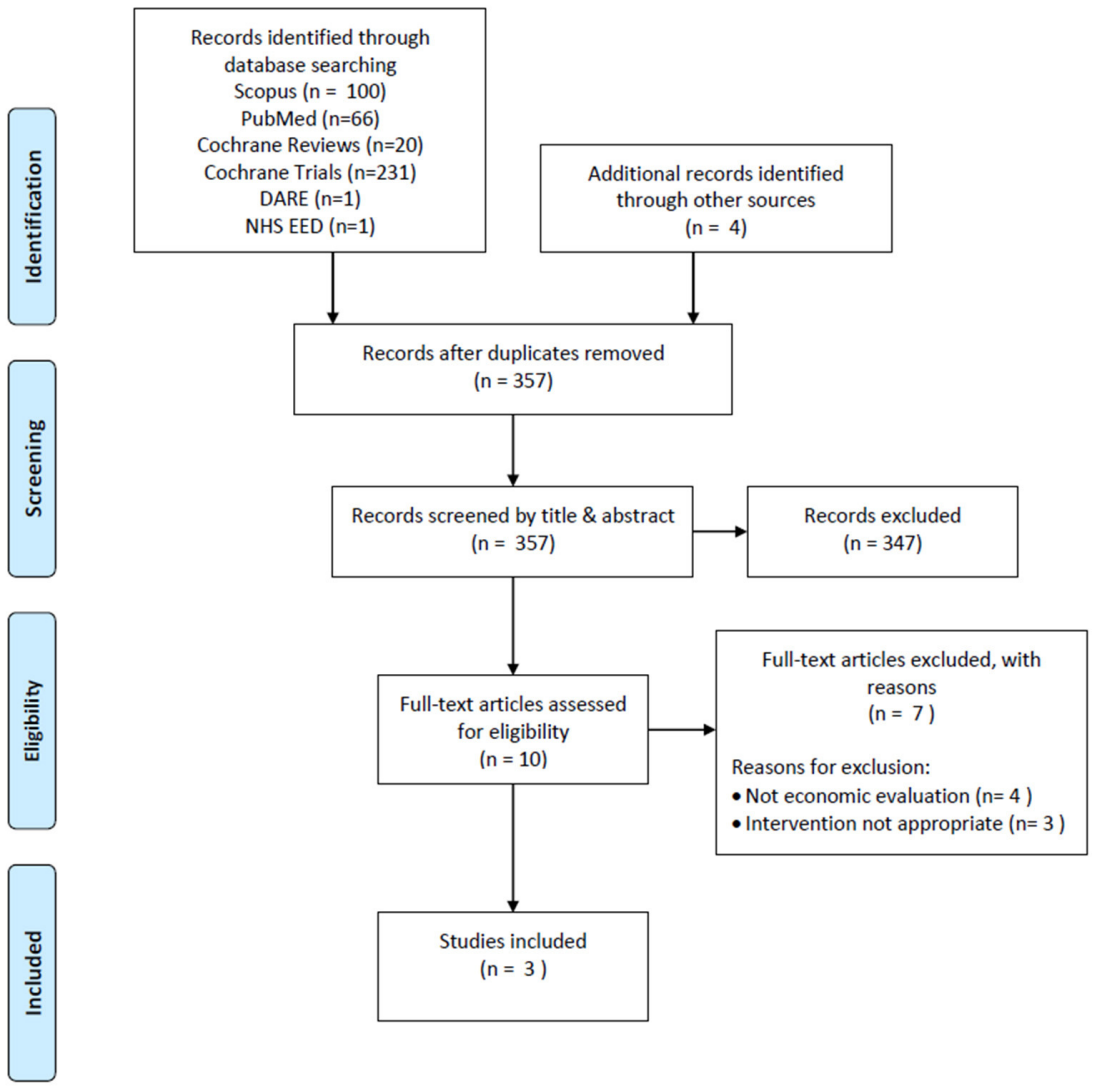
PRISMA flow chart.

The database searches returned a total of 419 publications, while 4 additional records were identified from reference scanning. Out of these, 66 titles were removed as duplicates, resulting in 357 records to be screened by title and abstract, of which 347 were removed as not meeting the inclusion criteria. Thus, 10 records were subjected to full-text review leading to 3 publications eligible for inclusion. The reasons for excluding 7 articles after full-text review were that they were not economic evaluations (*n* = 4) and the intervention was not appropriate (*n* = 3) (Figure 1).

Out of the three eligible studies, one (14) focused on an intervention that was delivered through video consultations, while the other two (15, 16) assessed eHealth interventions. Only one out of three included studies referred explicitly to palliative care as the intervention of interest (14), while the other two employed specific symptom management interventions (15, 16). The details of all the included studies are presented in Table 2.

**Table 2:** Table of included studies.

**Study**	**Country**	**Study design**	**Type of economic evaluation**	**Perspective**	**Population**	**Intervention**	**Comparison**	**Outcomes**	**Conclusions**
Bradford et al. (14)	Australia	Retrospective	Cost minimization	Children's Health Service (state-wide service)	Children requiring palliative services (sample size not specified, only number of consultations n=95)	Home Telehealth Programme video consultations	Usual care (as either home visit or outpatient department consultations)	Assumed to be equivalent irrespective of mode of service delivery	HTP Home video consultations are cost-saving compared to usual care
Zhang and Fu (16)	USA	Randomized controlled trial	Cost-effectiveness (utility)	Society, healthcare providers and patients	Prostate cancer patients with persistent urinary incontinence (*n* = 336)	Biofeedback PFME + support group sessions Biofeedback PFME + one-to-one phone sessions.	Usual care (for both Usual Care-UC and Intervention-Non-Participating-INP groups)	HRQoL (EQ-5D), QALYs	Intervention is cost-effective compared to no intervention
van der Hout et al. (15)	Netherlands	Randomized controlled trial	Cost-utility	Societal	Survivors of head and neck cancer, colorectal cancer, breast cancer, and lymphoma (*n* = 625)	eHealth self-management application (Oncokompas)	Care as usual wait-list (late access to intervention)	HRQoL (EQ-5D), QALYs	The eHealth intervention has similar effectiveness and non-significantly lower costs compared to care as usual

The population of interest in all the included studies was cancer patients, pediatric (*n* = 1) and adults (*n* = 2). One of the studies (14) was conducted retrospectively and did not specify the number of patients included in the analysis, but reported only the total number of consultations that occurred in a 24-month period, while the other 2 studies (15, 16) were randomized clinical trials and, therefore, report actual number of participating patients. None of the studies involved terminal patients.

All included studies employed different types of economic evaluation, namely cost minimization (14), cost-utility (15), and cost-effectiveness (16). However, it should be noted that, although the study of Zhang and Fu (16) is reported as a cost-effectiveness analysis, in reality it would more appropriately be referred to as a cost-utility analysis given that the outcome measure of the study concerns utilities (health-related quality of life measure translated into QALYs).

The cost of the digital health intervention of interest was taken into account and explained in all the included studies. In particular, all 3 studies measured the costs incurred by the required equipment and function of the selected intervention, as well as travel costs for patients and staff, where applicable. Also, the time spent by healthcare staff on each group was accounted for in all the included studies, while productivity losses from the patient/caregiver perspective were measured in two out of the three studies (15, 16). The study which did not include productivity losses was the one with pediatric patients and justified this on the grounds that all the caregivers in the study had given up full-time work to care for their children (14).

### Studies' Summary

Bradford et al. used retrospective data to conduct a cost minimization analysis to assess whether home telehealth palliative consultations are less costly compared to home visit consultations and outpatient consultations (14). The method of cost minimization was selected as outcomes were assumed to be equivalent for all comparators. The results of the study showed that the mean cost of home telehealth consultations was lower than that of both its comparators with the home visit consultations incurring the highest costs mainly due to clinician's travel time costs.

Zhang et al. (17) conducted a cost-effectiveness analysis to assess whether an intervention including a computer-assisted biofeedback pelvic floor muscle exercise (PFME) combined with either a support group or telephone sessions was cost-effective compared to usual care (16). The study sample consisted of prostate cancer patients with persistent urinary incontinence. Both groups with biofeedback PFME demonstrated higher EQ-5D scores and lower costs compared to no intervention usual care, resulting in a lower total ICER.

van der Hout et al. assessed the cost-utility of an eHealth application for cancer survivors supporting symptoms self-management and prompting professional healthcare options when needed (15). The comparator of choice was a wait-list control group with patients who were to join the intervention at a later time. The results indicated that the intervention group had slightly higher incremental QALY gains and lower but not statistically significantly different incremental costs compared to the control group, while the sensitivity analyses showed favorable outcomes for the eHealth application.

## Discussion

Digital health interventions are an increasingly useful tool in the healthcare sector. The economic evaluation of such interventions is imperative in order for decision makers to be able to determine if they can be adopted and reimbursed by healthcare systems and third-party payers. The present systematic review revealed that economic evaluation has not yet become standard practice for digital health interventions in palliative care.

According to the literature, evidence on the cost-effectiveness of palliative care is not abundant. A recent systematic review (18) on economic evaluation of palliative care models identified only 5 relevant studies, all of which concluded that palliative care is cost-effective. However, none of those studies contained any intervention relevant to digital health.

Although it is recognized that the economic evaluation of digital health interventions follows the same general methodology as any other economic evaluation for devices and medicines, some differences are identified, especially in the identification of resource use and costing (19).

A comparative analysis of the included studies was not feasible, as their type of economic evaluation and their overall methodology were quite heterogeneous. However, their findings indicate that incorporation of digital technology in the provision of palliative care can provide benefits in terms of costs and outcomes. Overall, the methodological quality of the included studies was good (Table 3). Nevertheless, all included studies focused on specific populations and, thus, their applicability and generalizability to broader populations is limited. The choice of health-related quality of life as the main outcome measure in 2 out of 3 studies–the cost minimization one assumes equivalent effectiveness among the comparators and, thus, does not measure any outcomes–and especially the use of the same generic tool indicates a common denominator in the specific healthcare field, which has been also identified as the most important outcome domain in palliative care (20).

**Table 3:** Methodological quality of included studies.

	**Bradford et al. (14)**	**Zhang and Fu (16)**	**Van der Hout et al. (15)**
Q1. Was a well-defined question posed in answerable form?	Yes	Yes	Yes
Q2. Was a comprehensive description of the competing alternatives given?	Yes	Yes	Yes
Q3. Was the effectiveness of the programs or services established?	No	Yes	Yes
Q4. Were all the important and relevant costs and consequences for each alternative identified?	Yes	Yes	Yes
Q5. Were costs and consequences measured accurately in appropriate physical units?	Yes	Yes	Yes
Q6. Were costs and consequences valued credibly?	Yes	Yes	Yes
Q7. Were costs and consequences adjusted for differential timing?	n/a	n/a	n/a
Q8. Was an incremental analysis of costs and consequences of alternatives performed?	n/a	Yes	Yes
Q9. Was allowance made for uncertainty in the estimates of costs and consequences?	Yes	No	Yes
Q10. Did the presentation and discussion of study results include all issues of concern to users?	Yes	Yes	Yes

Based on the literature, one of the characteristics that does not allow for a consistent and generalizable assessment of digital health interventions, not only in the case of palliative care but also in other types of care, is the wide heterogeneity in methodology (21). In particular, there is a wide variety of types of interventions ranging from text messaging and videoconferencing to eHealth applications (22). Furthermore, the purpose of the interventions is not universal either, as they can be used for provision of information, education or symptom management, while they can also be designed not only for patients but also for caregiver support.

Despite the current scarcity of evidence on the cost-effectiveness of digital health interventions for palliative care, ongoing research projects are expected to provide important insight on the subject. Two randomized controlled trials are underway with inherently planned cost-effectiveness analyses; the MyPal project (23) which aspires to foster palliative care for patients with cancer through an eHealth application recording Patient Reported Outcomes (PROs), and the eSMART project (electronic Symptom Management using the Advanced Symptom Management System (ASyMS) Remote Technology) (24) which aims to assess the impact of real-time remote symptom monitoring devices on morbidity and prevention of unplanned admissions.

Overall, the results of the present systematic review indicate that integrating digital health interventions in palliative care has the potential to be cost-effective. However, it highlights the need to develop a robust and consistent methodological framework in order to foster the implementation of high quality economic evaluation research.

### Strengths and Limitations

To our knowledge, this is the first systematic review that aimed to identify the existing literature on economic evaluation of digital health interventions for palliative care.

One of the main limitations of the present study is the possibility of not identifying all the existing publications on the subject due to limited access to databases.

## Data Availability Statement

The original contributions presented in the study are included in the article, further inquiries can be directed to the corresponding author.

## Author Contributions

PN, EP, and KA contributed in the study design and construction of the search strategy. PN and KA conducted the screening of the studies, while EP acted as an independent reviewer to resolve any disputes in the process. PN extracted the data of the analysis and EP corroborated them. PN wrote the first draft of the manuscript, while EP and KA added further elements in the text. All authors contributed to manuscript revision, read, and approved the submitted version.

## Conflict of Interest

The authors declare that the research was conducted in the absence of any commercial or financial relationships that could be construed as a potential conflict of interest.

## Publisher's Note

All claims expressed in this article are solely those of the authors and do not necessarily represent those of their affiliated organizations, or those of the publisher, the editors and the reviewers. Any product that may be evaluated in this article, or claim that may be made by its manufacturer, is not guaranteed or endorsed by the publisher.
